# A pilot QI primary care practice program to help reduce infant mortality risks

**DOI:** 10.1186/s40621-020-00252-3

**Published:** 2020-06-12

**Authors:** Michael A. Gittelman, Kristen Fluitt, Samantha Anzeljc, Arun RajanBabu, Adam C. Carle, Melissa Wervey Arnold, E. Melinda Mahabee-Gittens

**Affiliations:** 1 Division of Emergency Medicine, Cincinnati Children’s Hospital Medical Center, 3333 Burnet Avenue, MLC 2008, Cincinnati, OH 45255 USA; 2 Ohio Chapter of the AAP, 94-A Northwoods Blvd, Columbus, OH 43235 USA; 3 Department of Pediatrics, College of Medicine, University of Cincinnati, Cincinnati, OH USA; 4 Ohio Colleges of Medicine Government Resource Center, 150 Pressley Hall, 1070 Carmack Road, Columbus, OH 43210 USA; 5 James M Anderson Ctr for Health Systems Excellence, Cincinnati Children’s Hospital Medical Center, 3333 Burnet Avenue, Cincinnati, OH 45255 USA; 6 Department of Psychology, College of Arts and Sciences, University of Cincinnati, Cincinnati, OH USA

**Keywords:** Infant mortality, Office-based counseling, Smoking cessation, Quality improvement

## Abstract

**Background:**

Tobacco smoke exposure (TSE) and inappropriate sleep position/environments contribute to preventable infant deaths. The objective of our quality improvement (QI) program was to increase primary care provider (PCP) screening and counseling for TSE and safe sleep risks at well-child visits (WCVs) and to assess caregiver behavior changes at subsequent visits.

**Methods:**

Pediatric practices, recruited from the Ohio Chapter, American Academy of Pediatrics’ database, self-selected to participate in this TSE and safe sleep PCP QI program. At every WCV over a 10-month period, caregivers with children < 1 year old were to be screened and counseled by providers. Caregiver demographics, TSE, and safe sleep practices were assessed. Individual PCP results were paired with subsequent family screening tools at follow up visits to determine changes in TSE and safe sleep practices. Differences in frequencies were determined and paired t-tests were used to compare means.

**Results:**

Fourteen practices (60 providers) participated; 7289 screens were completed: 3972 (54.5%) initial screens and 1769 (24.3%) subsequent WCV screens. Caregivers on the initial screen were primarily white (61.7%), mothers (86.0%) with public insurance (41.7%). Within the first month after QI program initiation, PCPs TSE screening was during 80% of WCVs, which increased to > 90% by end of the QI program. A total of 637 /3953 (16.1%) screened positive for home TSE on the initial visit: 320/3953 (8.1%) exposed by at least the primary caregivers, and 317/3953 (8.0%) exposed by a home adult smoker (not the identified caregiver). Of caregivers receiving smoking counseling with subsequent follow-up WCV (*n* = 100), the mean number of cigarettes smoked daily decreased significantly from 10.6 to 4.6 (*p* = 0.03). Thirty-four percent of caregivers (34/100) reported they quit smoking at their second visit. A total of 1072 (27%) infants screened at risk for inappropriate sleep position or environment at their initial visit. Of these at-risk infants whose caregivers received safe sleep counseling, 49.1% practiced safer sleep behaviors at follow-up.

**Conclusions:**

PCPs participating in a QI program increased screening at WCVs for infant mortality risks. After counseling and providing resources about TSE and safe sleep, many caregivers reported practicing safer behaviors at their next WCV.

## Background

Infant mortality rates (IMR) are widely used as a marker for the overall health of a society (Infant mortality n.d.-a). IMR has served as a crude indicator of community health, poverty, and the availability of quality health services (http://www.amchp.org/programsandtopics/data-assessment/InfantMortalityToolkit/Documents/Why%20Focus%20on%20IM.pdfn.d.). In the US, the two leading causes of infant mortality are congenital malformations and preterm/low birthweight gestation (Infant Mortality n.d.-b). Although prenatal care, including taking necessary medications (e.g. prenatal vitamins and folic acid) and avoiding alcohol, cigarettes and drugs, could reduce these common causes, focusing on modifiable parental behaviors after birth, like tobacco smoke exposure (TSE) and sleep-related deaths, can also reduce IMR. In particular, second hand and third hand TSE from parents and caregivers has been consistently linked to an increased risk of many conditions causing higher IMRs, such as bronchiolitis, lower respiratory illnesses and sudden infant death syndrome (SIDS) (Wang et al. 2015; Jones et al. 2011; Dept US. Of health and human services 2006). In addition, sudden unexpected infant deaths (SUIDS), the third leading cause of infant mortality in the U.S., can be prevented by following recommended infant sleep guidelines (American Academy of Pediatrics Task Force on Sudden Infant Death Syndrome 2011).

When primary care providers (PCPs) screen families with children < 1 year old for injury risk and counsel them about safety recommendations, 65% made at least one behavior change in their home, and more than 40% changed all risky behaviors (Gittelman et al. 2018). In one literature review, 18 of 20 studies showed varying increases in safety knowledge and behaviors and decreases in childhood injury rates after PCP counseling (Bass et al. 1993). Similarly, pediatricians have the opportunity to screen families for TSE as a way to reduce TSE-related illnesses and SIDS (Hall et al. 2015). Counseling caregivers who smoke and referring them to a smoking cessation program has resulted in reductions in continued TSE to the child (Nabi-Burza et al. 2019; Drehmer et al. n.d.).

Quality improvement (QI) methodology in the pediatric office setting has been successful in helping primary care providers (PCPs) improve their clinical practice and instigate family behavioral change. When PCPs implement tools through QI initiatives, families are more consistently screened for health risks and provided with individualized, tailored counseling, that can result in greater behavioral change than population-based, generic handouts (Nansel et al. 2002). Providers are becoming more engaged in office QI efforts in general since QI initiatives have become a part of their physician maintenance of certification (MOC). Practice efficiency, increased staff retention and satisfaction have also been cited as successes of QI work in an office practice (Wolfson et al. 2009). These QI initiatives are typically easy to implement into a busy practice environment in a short time period (Gittelman et al. 2015).

To improve PCP screening and counseling regarding infant TSE and safe sleep, we developed a QI program to be used at infant well-child visits (WCV). The primary purpose of this QI program was to implement a standardized screening tool at every WCV, for families with children < 1 year old, to assess TSE and infant sleeping risks and encourage provider counseling, when warranted. Screens at subsequent WCVs were analyzed to evaluate self-reported behavior changes by families who screened at-risk and received guidance from their PCP.

## Methods

### Study design

We conducted a prospective study of TSE exposure and infant safe sleep practices pre- and post-targeted pediatric provider counseling at WCVs. This PCP QI program was conducted between January 1, 2018 and October 31, 2018. The data for this study were collected during our QI program with pediatric practices. The aim of the QI program was to provide PCPs with a standardized home TSE and infant safe sleep screening tool to be used with every family that has children < 1 year old during their WCV. The main goal of the QI program was to have PCP’s screen > 90% of families for TSE and safe sleep risk at WCVs and to counsel/provide resources for > 90% of at-risk families by the end of the QI collaborative. As pediatric providers implemented tools and counseled caregivers who screened at-risk for TSE/ unsafe sleeping patterns, self-reported behavior changes by families at WCVs were assessed. Approval of the study protocol was obtained by the Ohio State Behavioral and Social Science Institutional Review Board (IRB) prior to study initiation.

### Setting

Sixteen primary care practices were recruited from the Ohio Chapter, American Academy of Pediatrics (Ohio AAP) membership database and volunteered to participate. The number of practices were capped at sixteen, due to resources available for the study. Mailings and postings in newsletters were sent to members notifying them about participation. Core QI teams were chosen by each participating practice consisting of a physician leader, a nurse/nurse practitioner or medical assistant, and an administrative staff/office manager. The QI program was similar to the Institute of Healthcare Improvement (IHI) Breakthrough Series Collaborative (Institute for Health Care Improvement (IHI) 2003). Core teams participated in a pre-work conference call outlining the requirements for the program. Core team members were expected to educate the other participating practitioners from their practice on the learning collaborative content. All physician members in each practice that submitted data received American Board of Pediatrics (ABP) Maintenance of Certification (MOC) IV credit for participation through the Ohio AAP.

### Screening tool development

The screening tool was developed by experts affiliated with The Ohio State University, the Ohio Colleges of Medicine Government Resource Center (GRC), and tobacco and injury prevention experts from the Ohio AAP. The tool was comprised of six components: patient and family demographics, home TSE behavior and infant sleep screen, location and self-reported amount of TSE in the home, past attempts to quit smoking, motivation to quit smoking, and counseling and resources provided by the PCP (Additional File) (Biener and Abrams 1991a). Demographic questions included the child’s date of birth and race/ethnicity, home zip code, date of visit, caregiver’s age and relationship, and family insurance type. Caregivers were screened to assess their infant’s current TSE and sleep practices in the home setting. Parental consent for participation was not required as every participating practice implemented the screening tool into their daily routine in a quality improvement initiative. If there was no infant TSE, no further questions were asked of the caregiver, and the PCP was handed the tool in order to complete their section of the form. TSE was assessed by asking the caregiver the number of cigarettes, e-cigarettes, or cigars smoked by themselves or other household residents daily in the past 7 days. Once all the screening questions were completed, PCPs were instructed to provide families with counseling and resources developed by the QI team that included specific TSE reduction, smoking cessation or infant safe sleep talking points. If other smokers were noted in the home, resources were provided to the caregiver only, since the other person was not present for the visit to receive counseling.

Data on the frequency and types of discussions and resources provided were captured at the encounter on the screening tool. All caregivers willing to quit smoking were then offered a referral to the Ohio Quitline (Ohio Department of Health n.d.). Only offering referral was captured, not if a referral was actually sent. All tools were completed on paper, and electronic medical records were not used for data collection since there was no consistency in electronic records available to providers. If caregivers struggled with answering questions on the tool, assistance was provided to them by office staff. To examine one balancing measure, we quantified the duration of time it took for caregivers to complete the survey during the WCV. Finished tools were scanned and data was automatically entered into a REDCap™ database for the GRC and data analyst team to monitor QI goals and assess caregiver behavior change.

### QI performance goals

Two main performance goals were tracked and discussed with practices on monthly calls:

1. > 90% of caregivers presenting to a PCP office for a WCV will be screened for infant safe sleep practices and TSE in the home setting.
2. > 90% of caregivers who screen positive for smoking or inappropriate safe sleep practices will receive counseling to quit smoking (including a referral to Ohio Quitline if willing) and to practicing infant safe sleep.

### Quitline

#### Data collection

##### Baseline

The lead pediatric providers responded to a pre-QI program survey prior to a one-day learning collaborative. All data for this survey were captured on SurveyMonkey®, and summaries were analyzed prior to participation. The survey included assessments of practice demographics (e.g. number of infants seen monthly at WCVs, practice setting, payer breakdown), current method used to collect patient data (e.g. electronic, paper, etc.), smoking screening and resources provided to families, and provider QI experience. The purpose of the baseline screen was to assess general practice behaviors only; individual provider charts were not reviewed.

### Learning collaborative and action period

At least one core team member from each practice attended a one-day face-to-face learning session held on January 26, 2018. Learning session objectives were to educate team members about the importance of discussing smoking cessation and infant safe sleep at a WCV, principles of QI methodology, including how to conduct Plan-Do-Study-Act (PDSA) cycles, how to implement the proposed screening tool into practice, and how monthly data should be collected and reported (Gittelman et al. 2018).

After the one-day learning session, caregivers presenting with children < 1 year old for a WCV were to be screened using the given tool during the 10-month collaborative. Tools were kept in a secure location by practices and were scanned weekly for direct data entry into the REDCap™ database for GRC analysis. Three items of protected health information (PHI) was used to match individuals for follow-up visits (date of birth, date of visit, home zip code); PHI was not used in any other data analysis. All tools were reviewed on a monthly basis to ensure that QI metrics were being met by practices. Also, practices provided the number of children < 1 year old seen by each provider for a WCV to capture the percent of monthly screens completed. Changes in providers’ screening and counseling about TSE, smoking cessation and infant safe sleep over time were determined and presented individually and in aggregate during monthly action period calls. This provider level data was not blinded during these calls in order to increase inter-practice level competition and to help share successes and areas for improvement. When addressing appropriate and inappropriate responses at repeat screens, previous screening was not provided to the PCP to prevent bias from pediatric provider counseling.

### Analysis

Families who completed the screening tool on subsequent WCVs were matched with their prior WCV to determine self-reported behavior changes after discussion was provided by the PCP. Repeat screening tools completed by a different caregiver were omitted from the behavior change analyses due to a potential lack of reliability when the tool is used with different caregivers. Also, only the second WCV conducted at least 1 week after the initial visit was used in the follow-up analysis. Third or fourth visits were excluded since there were too few data points to make assessments about change at these visits, and we were mainly determining behavior change after counseling at the initial visit. Differences in frequencies were determined and paired t-tests were used to compare means to analyze behavior changes. Because our interest was solely in whether caregivers decreased smoking, we used a one-sided t-test. To determine change in behavior regarding home TSE at the subsequent visit, caregivers had to screen positive in the first visit and be counseled about smoking cessation. For other smokers in the home, we did not include only those who received counseling, since the other smoker was not at the first WCV and did not personally receive counseling.

## Results

Sixteen practices were recruited and completed the baseline survey. Two practices left the collaborative after completing baseline intake, leaving 14 practices with 60 pediatric providers to participate in the action period of the QI program. All providers remained active during the collaborative, and no practitioner dropped out once data collection began. At baseline, for the 14 participating practices, most of the lead providers self-identified their practice as hospital-affiliated (7 (50%)) and in a suburban setting (9 (64.3%)). The majority used electronic medical records (12 (85.7%)). Their average monthly volume for children < 1 year old presenting for a WCV was approximately 200, and the insurance providers of their patients were primarily Medicaid 48% (private payer only 45.9%). Prior to the learning collaborative, 9 practices (64.3%) indicated they routinely screened families with infants for home TSE; however, only 1 (7.1%) provided handouts on why to quit, and 3 (21.4%) offered phone numbers or websites to assist caregivers with smoking cessation. Most of the practices reported to participate in at least one previous QI initiative (12 (85.7%)).

A total of 7289 screens were completed during the action period: 3972 (54.5%) initial screens, 1769 (24.3%) at next WCV, and 1548 (21.2%) repeat screens at other WCVs. We focus on the first and second WCVs in these analyses (*n* = 5741). The majority of caregiver respondents on the initial screen were non-Hispanic Caucasian (2425 (61.7%)), mothers (3400 (86.0%)), and 1625 (41.7%) had public insurance (Table 1). The mean age of the child at the initial visit was 124.8 days (median 68 days, SD 123.3). On the initial screens, 637/3953 (16.1%) caregivers reported their infant had TSE: 320 (8.1%) primary caregivers 173 (4.4%) caregiver and someone else in the home), and 317 (8.0%) reported at least one other smoker in the home, but not the caregiver. Of the 320 smoking caregivers, 271 (84.2%) reported they smoked outside the house, and 36 (11.3%) inside the home, but in another room. Among cigarette smokers, the median number of cigarettes smoked per day in the last week was 9, and the mean was 10 (SD = 15.8). When smoking caregivers were asked about their readiness to quit on the initial screen, 51 (18.3%) reported they were not willing (answered 0–2 on readiness scale), and 72 (17.5%) stated that others in their house wouldn’t be willing. In addition, 1072 (27.0%) caregivers reported their child slept in an inappropriate location or environment. For the balancing measure of extra time needed during the WCV to conduct the TSE and safe sleep screening, on average, surveys only took approximately 5–7 min to complete by caregivers.

**Table 1:** Demographics of initial survey responses by families at well-child visits

***N*** = 3972	Description	Number (%)
**Race**	**Caucasian**	2425 (61.0)
	**African American**	783 (19.9)
**Age of Caregiver**	**15–25 years**	1106 (28.1%)
	**26–35 years**	2286 (58.0%)
	**>36 years**	550 (13.9%)
**Relationship**	**Mother**	3400 (86.0)
	**Father**	441 (11.2)
**Insurance type**	**Medicaid/Public Insurance**	1625 (41.7)
	**Private**	1684 (43.2)

**Fig. 1:**
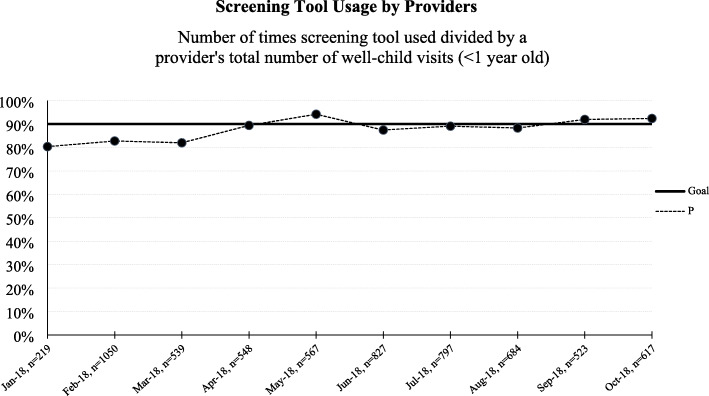
Uptake of screening tool use per month

Prior to the learning collaborative, practice leaders reported only 10/16 (62.5%) practices routinely screened for smoking at WCVs, and only 1/16 (6.3%) practice reported providing resources. Within the first month, more than 80% of participating providers documented screening for home TSE, and these efforts increased to > 90% by the end of the collaborative (Fig. 1). In addition, pediatricians more consistently (> 70% of the time) providing smoking caregivers resources and referrals during the action period. The most common PDSA cycles implemented by practices to encourage change were completed in the first 3 months of the collaborative and included: pre-visit team planning, visual reminders for staff, and documentation by extended providers on the team.

Slightly more than 94 % of smoking caregivers (252/267) received counseling from their PCP. One hundred smoking caregivers received counseling and had a repeat visit. The average length of time between visits among this group was 74 days (Median = 63). Among this group, the mean number of cigarettes dropped significantly from 10.6 to 4.6 from one visit to the next (*p* = 0.03). In addition, 34/100 (34%) reported they were not currently smoking at their subsequent visit. Unfortunately, 2.3% of non-smokers at baseline had started smoking at follow-up. Of the 1072 families who screened at-risk for infant safe sleep, 860 (80.2%) were counseled by their PCP. For the 401 caregivers that screened at-risk and were counseled at the initial visit, 197 (49.1%) changed their behavior at follow-up. (Table 2)

**Table 2:** Counseling and behavior change for TSE and safe sleep

	Screened + or At-risk Initially n/N (%)	Received Counseling n/N (%)	Change in Behavior n/N (%) (Reduction in home TSE or Change in sleep practices)
**TSE Caregivers**	320/3953 (8.1)	252/267 (94.4)^a^	51/95 (53.7)^b^
**TSE Exposure to Others (not Caregiver)**	317/3953 (8.0)	234/252 (93.0)^a^	81/197 (58.9)^b^
**Safe Sleep**	1072/3959 (27.1)	860/1072 (80.2)	197/401 (49.1)^b^

^a^Column 2 denominator not equal to column 1 numerator because of missing data

^b^Column 3 denominator not equal two column 2 numerator because of loss to follow-up

## Discussion

This study demonstrates providing PCPs with tools and resources to use in practice through QI initiatives increases screening and counseling parents with infants about smoking cessation and safe sleep practices at WCVs. By making the tools easy to use, practice changes can be implemented quickly and sustained over time. In addition, when pediatricians counsel caregivers about TSE and sleep risks, significant behavior change is reported at subsequent visits.

The primary goal of this program was to improve consistent PCP screening of caregivers with children < 1 year old in the home for TSE and sleep-related injury risk at WCVs and to increase appropriate counselling. This work supports previous work showing QI methodology can improve the delivery of prevention anticipatory guidance for caregivers in the pediatric office setting (Bordley et al. 2001). In general, office changes from QI work to reach the desired effect can vary depending on changes sought and effort by the team. Incorporating our TSE/infant safe sleep screening tool into office flow was relatively quick, within the first 1–2 months. This rapid effect was likely due to our prescreening process and tutorial of key team members prior to start of the program, the easy to use, scan able tool and the emphasis of this work by the practice. Using similar methodology with other programs, our team has shown similar results of improving screens and counseling early within the intervention (Gittelman et al. 2015).

Screening our population identified risks similar to other caregiver study populations. Combining caregivers who smoke with other family members who smoke identified 16% of children < 1 year old at-risk for TSE at their WCV. In 2017, the Center for Disease Control reported a significant reduction in US adult smoking rates to 14% (Preidt n.d.). Other studies have found slightly higher current smoking rates of 25% within primary care practices (Nabi-Burza et al. 2019). Our sample had lower primary caregiver smoking rates, possibly due to caregivers recently completing pregnancy and the child’s young age. Also, the average number of cigarettes smoked per week and the mean motivation to quit smoking on a scale of 0–10 was similar to other studies encouraging parents to quit smoking (Biener and Abrams 1991b). Caregiver responses of inappropriate infant sleep was 27% in our study; a number slightly lower than the 48% at-risk determined in our previous injury prevention study (Gittelman et al. 2018). This could be due in part to the dissemination efforts of the 2017 AAP infant safe sleep recommendations to providers and the public (American Academy of Pediatrics Task Force on Sudden Infant Death Syndrome 2011).

For caregivers who smoked and received counseling by their child’s pediatrician, a reduction of daily cigarettes smoked over the past week was reported at their next WCV. Smoking cessation counseling in pediatric offices has been shown to be effective in the past. Within our sample, we found a greater reduction of reported TSE compared to other studies, which could be due to the young age of infants in this study (Hawkins and Berkman 2011; Zhang et al. 2015). This suggests counseling caregivers of infants may entice greater behavior change than counseling parents with older children, but more research is needed. Unfortunately, on repeat screens, we did find 2.3% of nonsmokers started to smoke by their next visit. This would indicate screening for TSE should be recommended at every visit, even if a caregiver screens not at risk previously. In addition, a significant number of families who screened at risk for sleep-related injury and received PCP counseling, practiced safer behaviors at their return visit. These results are similar to results of our previous QI work implementing an injury prevention screening tool in the primary care setting (Gittelman et al. 2018).

This study is not without some limitations. First, respondents may inappropriately state a change in their behavior due to exposure bias, as they were screened with the same tool at a previous office visit. However, re-visits occurred at the child’s next WCV, which was typically at least 2 months after the initial visit and memory of all specific responses from their previous screen is unlikely. The responses by families to elicit behavior change were self-reported thus social desirability and recall biases, especially in the healthcare setting, could have occurred. Another limitation is some families did not have a follow-up visit, thus subsequent behavior change could not be assessed. Also, the complete number of screens performed by each practitioner was not determined. We encouraged practices to screen all children < 1 year old who were seen by the participating PCP; however, we were not able capture all of this information during the collaborative. In addition, baseline data about practice behaviors were captured through SurveyMonkey® instead of by reviewing providers medical records. Because the main purpose of the study was caregiver behavior change and the QI component was practice change during the collaborative, the study team felt the time and cost of reviewing records for baseline practices was not necessary. Lastly, PCPs had a vested interest during the action period to show change as they were participating to receive MOC credit. However, the PCPs were evaluated on their ability to address risky behaviors assessed on screen, not the positive change by the caregiver on subsequent visits. In addition, PCPs did not have immediate access to the families’ initial screens, so these responses were not known at the next visit.

## Conclusions

Infant mortality is a significant problem in the United States. Reducing infant TSE and having families follow AAP-recommended infant safe sleep practices can help to reduce the high IMR. PCPs can encourage families to change their behaviors if they consistently screen caregivers for risk, counsel them on best practices and provide them with resources. In this study, a TSE and safe sleep QI program demonstrated an increase in caregivers reporting a reduction in TSE and more appropriate infant sleep positions and environments based on physician recommendations. PCPs should standardize their practices to improve screening families for TSE and infant sleep risks and counsel, when indicated.

## Supplementary information

**Additional file 1.** Screening tool used at well-child visits for children 0–1 year of age.

## Data Availability

More in depth data and materials are available upon request.

## Abbreviations

AAP: American Academy of Pediatrics
ABP: American Board of Pediatrics
GRC: Government Resource Center
IHI: Institute of Healthcare Improvement
IMR: Infant mortality rate
IRB: Institutional Review Board
MOC: Maintenance of Certification
PCP: Primary care provider
PDSA: Plan-Do-Study-Act
PHI: Personal health information
QI: Quality improvement
SIDS: Sudden Infant Death Syndrome
SUIDS: Sudden Unexpected Infant Deaths
TSE: Tobacco Smoke Exposure
US: United States
WCV: Well child visit
