# Supplementary material for: A pilot QI primary care practice program to help reduce infant mortality risks
Source: Inj Epidemiol. 2020 Jun 12;7(Suppl 1):25. doi: 10.1186/s40621-020-00252-3 (PMC7291417; doi:10.1186/s40621-020-00252-3)
Supplement: Supplementary file 1 — Additional file 1. Screening tool used at well-child visits for children 0–1 year of age. [file 40621_2020_252_MOESM1_ESM.pdf]

Smoke Free for Me

American Academy of Pediatrics
DEDICATED TO THE HEALTH OF ALL CHILDREN™
Ohio Chapter

Have you completed this form at a previous visit?
○ No ○ Yes (If yes, only answer Q 1, 2, 3 and move on to Q 8)

1) Baby's date of birth (MM/DD/YYYY): ____ /____ /________

2) Your home zip code: ___________

3) Today's date: ____ /____ /________

4) Baby's race (Please completely fill in all circles that apply):

○ African American or Black ○ White ○ Hispanic or Latino
○ Asian ○ American Indian or Alaska Native ○ Other:____________________

5) Your age: ○ 15-20 ○ 26-30 ○ 36-40 ○ 46-50 ○ 56-60
○ 21-25 ○ 31-35 ○ 41-45 ○ 51-55 ○ 61+

6) What is your relationship to the baby (Please fill in only one circle)?

○ Mother ○ Foster Parent ○ Aunt/Uncle ○ Other:
○ Father ○ Grandparent ○ Brother/Sister ________________

7) What kind of health insurance do **YOU**, the caregiver of the baby, currently have? (Please fill in all circles that apply.)

○ Private health insurance ○ Other health insurance: ○ I do not have health insurance now
○ Medicaid/Medicare ____________________

8) I **ALWAYS** place my baby to sleep for a nap or at nighttime, on their back, in their own crib, **AND** with **NOTHING ELSE** in the crib. ○ YES ○ NO

9) Do **YOU** currently smoke or vape inside or outside of the baby's home? ○ YES ○ NO

10) Do **OTHERS** who live in the home with the baby currently smoke or vape inside or outside of the home? ○ YES ○ NO

If you answered "No" to both questions 9 and 10 **STOP** the survey. Otherwise, continue to the next question.

11) On a scale from 0-10, what number shows your thoughts about quitting? ○ I do not smoke/vape

No thought of quitting now		Should consider quitting someday		Should quit but not quite ready		Thinking about cutting down or quitting		Have cut down and seriously considering quitting		Ready to quit
0	1	2	3	4	5	6	7	8	9	10
○	○	○	○	○	○	○	○	○	○	○

12) On a scale of 0-10 what number shows your thoughts about talking to someone who lives in the home who smokes or vapes about quitting?

○ Others living in the home with the baby do not smoke/vape

No way I can		If I talk to them, they still won't quit		Thought of it before		I would consider talking to them		I've talked to them before and want to do more		Tell me what I need to do
0	1	2	3	4	5	6	7	8	9	10
○	○	○	○	○	○	○	○	○	○	○

13) How many other people who live in the house have smoked or vaped inside your home or car where the baby rides in the past 7 days?

○ No one else smokes ○ 1 ○ 2 ○ 3 ○ More than 3

14) How much have you or others living in the home smoked or vaped in the past 7 days? For each product please fill in 00 if you or others do not smoke that product.

Example A: 1 | 2; B: 0 | 5	You		Others	
	**A)** Please write in the two boxes below the number of cigarettes ***you*** smoke per day in the past week. For **example**, if you smoke 10 cigarettes per day, you would enter **1 in box 1** and **0 in box 2**:		**B)** Please write in the two boxes below the number of cigarettes ***all others living in the home with the baby*** smoke per day in the past week. For **example**, if others smoke 5 cigarettes per day, you would enter **0 in box 1** and **5 in box 2**:	
	Box 1	Box 2	Box 1	Box 2
**Cigarettes**				
	**C)** Please write in the two boxes below the **number of times per day *you*** smoke cigar/black & milds in the past week.		**D)** In the two boxes below, write the average **number of times per day** that ***others living in the home with the baby*** smoke cigars/black & milds.	
	Box1	Box 2	Box 1	Box 2
**Cigars/ Black & Milds**				
	**E)** Please write in the two boxes below the **number of times per day *you*** vape/use e-cigarettes/hookah in the past week.		**F)** In the two boxes below, write the average **number of times per day *others living in the home with the baby*** vape/use e-cigarettes/hookah.	
	Box 1	Box 2	Box 1	Box 2
**Cicarettes/Vape Pens/Hooka**				

15) Recently, (last few weeks), have you or others living in the home tried to quit or have smoked/vaped less around the baby? (**Please fill in all circles that apply**)

I have...	Generally, others in the house have...
○ Never smoked/vaped	○ Never smoked/vaped
○ Tried smoking/vaping less around the baby	○ Tried smoking/vaping less around the baby
○ Tried to quit smoking/vaping	○ Tried to quit smoking/vaping
○ Successfully quit smoking/vaping	○ Successfully quit smoking/vaping
○ Made NO changes to my smoking/vaping amount or where I smoke/vape	○ Made NO changes to my smoking/vaping amount or where I smoke/vape

STOP SURVEY

Smoke Free for Me – Healthcare Provider Data MD

		Step I. ADVISE		Step II. Assist		Step III: Arrange
	Nurse/MA to Complete	MD to Complete				
If "Yes" response to Q8	MD Should Discuss Safe Sleep?<br>○ Yes<br>○ No	MD Addressed Safe Sleep<br>○ Yes ○ No ○ N/A		Provider Initials		
If "Yes" response to Q9 / Question 13: 2-10	MD Should Discuss Caregiver Smoking/Vaping?<br>○ Yes (Handouts #: 1,4)<br>○ No<br>MD Should Refer to Quitline<br>○ Yes (Handouts #: 2,6,7)<br>○ No (Handouts #: 3)	MD Attempted to Discuss Caregiver Smoking Based on Readiness to Change<br>○ Yes ○ No ○ N/A		Caregiver Smoking Resources Offered?<br>○ Yes ○ No ○ N/A		MD Offered Referral to Quitline<br>○ No – Declined<br>○ No – Other<br>○ Yes ○ N/A
If "Yes" response to Q10	MD Should Discuss Other(s) Living in the Home Smoking/Vaping<br>○ Yes (Handouts #: 2,7)<br>○ No	MD Attempted to Discuss Other(s) Living in the Home Smoking/Vaping	○ Yes<br>○ No<br>○ N/A	Other(s) Living in the Home Resource Packet/(Handouts #2 & 7, or 3) Offered?	○ Yes<br>○ No<br>○ N/A	
